# Thermo‐Kinetic Design of Magnesiothermic Reduction for Morphology‐Retaining Porous Si@C Anodes

**DOI:** 10.1002/smll.75004

**Published:** 2026-07-31

**Authors:** Chaeheon Woo, Yeongjin Kim, Xue Dong, Byung Joo Jeong, Xiaojie Zhang, Hyeon‐Seok Bang, Kyung In Kim, Minjae Kim, Donghyeon Lee, Junyoung Mun, Hak Ki Yu, Jae‐Young Choi

**Affiliations:** ^1^ School of Advanced Materials Science and Engineering Sungkyunkwan University Suwon Republic of Korea; ^2^ SKKU Advanced Institute of Nanotechnology (SAINT) Sungkyunkwan University Suwon Republic of Korea; ^3^ Clean Energy Research Center Korea Institute of Science and Technology Seoul Republic of Korea; ^4^ KIST‐SKKU Carbon‐Neutral Research Center Sungkyunkwan University Suwon Republic of Korea; ^5^ SKKU Institute of Energy Science and Technology (SIEST) Sungkyunkwan University Suwon Republic of Korea; ^6^ Department of Materials Science and Engineering & Department of Energy Systems Research Ajou University Suwon Republic of Korea

**Keywords:** carbon exoskeleton, magnesiothermic reduction, porous Si@C, silicon anodes, supply‐consumption balance

## Abstract

Silicon (Si) is a promising anode material for replacing graphite, but nano‐sized Si suffers from low electrode density, whereas micro‐sized Si undergoes severe volume expansion and structural degradation, limiting cell‐level performance. Magnesiothermic reduction, a conventional route for converting silica precursors into Si, often causes impurity formation and morphology collapse because Mg supply is not balanced with the surface reaction rate. A thermo‐kinetic process model is introduced to guide process design by balancing Mg supply and surface consumption during reduction. By tuning reaction conditions to maintain this balance, Stöber SiO_2_ is converted into monodisperse spherical porous Si without aggregation. A carbon exoskeleton is introduced before acid etching to preserve the fragile porous framework during purification. Under the optimized condition, the resulting Si@C delivers a reversible capacity of 1571.1 mAh g^−1^ at the 100th cycle with an initial coulombic efficiency of 74.2%. The porous architecture and carbon exoskeleton mitigate mechanical degradation, while the monodisperse spherical morphology promotes more uniform electronic and ionic transport and reaction distribution within the electrode. These results demonstrate an effective process platform for producing morphology‐retaining porous Si@C through integrated thermo‐kinetic control and carbon‐assisted stabilization.

## Graphical abstract

Balancing Mg‐vapor supply and surface consumption during magnesiothermic reduction suppresses particle aggregation and preserves monodisperse morphology in porous Si@C spheres. The resulting architecture improves transport uniformity and reduces degradation within the electrode, leading to more stable high‐capacity cycling than aggregated counterparts and demonstrating a practical route to morphology‐retaining silicon anodes.

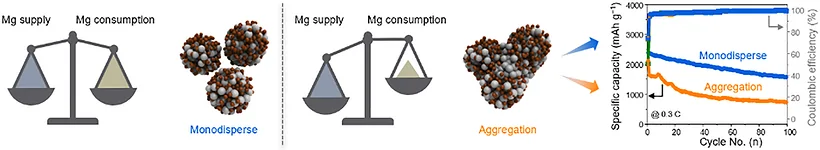

## 1 Introduction

Lithium‐ion batteries are increasingly required to deliver higher energy density and longer cycle life as electric vehicles and large‐scale energy storage systems become more widespread. Silicon (Si) has emerged as a leading candidate for next‐generation anodes because it offers a theoretical capacity nearly ten times (∼3579 mAh g^−1^) that of graphite and a low lithiation potential [1, 2]. However, not only the large volume expansion of Si and the resulting electrode fracture, but also the low electrode density of nanoscale Si has been identified as major factors limiting cell‐level implementation [3, 4, 5]. Nanoscale Si is advantageous in terms of reaction kinetics and mechanical buffering due to its high specific surface area and short diffusion length [6, 7], but its intrinsic porosity and the loosely packed particle bed with large interparticle voids lead to a low effective volume fraction of active material in the electrode [8]. To overcome these limitations, recent studies on Si‐based composite anodes have introduced surface/interface engineering, conductive coatings, and hierarchical or hybrid architectures to improve electrical conductivity, accommodate volume changes, and enhance Li‐storage kinetics [9, 10, 11, 12]. These efforts highlight that rational control of Si‐based particle architecture is important for balancing reaction accessibility and structural stability during cycling.

From this perspective, monodisperse spherical porous Si is a particle architecture that can simultaneously target morphology stability and electrode‐level reaction homogenization. A representative design strategy introduces nanoscale porosity inside the particles to shorten Li diffusion pathways and partially accommodate volume changes during lithiation/delithiation [13, 14], while controlling the overall particle size and packing behavior in the submicrometer‐to‐micrometer range as secondary spherical agglomerates [4]. More importantly, when the particle size distribution is narrow and the external morphology is well retained, electronic and ionic pathways can be formed more uniformly throughout the electrode, thereby mitigating local current hotspots, reaction inhomogeneity, and mechanically localized stress concentration [15, 16]. Accordingly, the key design challenge is not simply to introduce porosity, but to balance internal porosity with external morphology stability so that electrolyte accessibility, reaction homogeneity, and structural durability can be secured simultaneously.

Magnesiothermic reduction (MTR) of SiO_2_‐based precursors is a widely used approach for converting silica into Si, since it can proceed at relatively low temperatures and can be applied to various silica morphologies [17, 18]. However, in practical processes, non‐uniform Mg supply and local overheating caused by the exothermic reaction often lead to spatially heterogeneous reduction [19, 20, 21], where some regions are reduced either excessively or insufficiently. When Mg is supplied in excess, Mg_2_Si, which is known as an intermediate along the reduction pathway, forms in large amounts and remains only partially converted to Si, thereby promoting particle aggregation and morphology collapse [22]. Conversely, when Mg vapor supply is insufficient or its penetration and diffusion are limited, partially reduced structures containing Mg_2_SiO_4_ and residual SiO_2_ are formed, which degrade not only the phase purity of the final product but also its particle size distribution and morphology integrity [23, 24]. These issues act as fundamental barriers to reproducibly obtaining high‐purity, monodisperse spherical Si.

Therefore, to enhance the SiO_2_ to Si conversion while preserving a non‐aggregated, uniform spherical morphology, it is necessary to engineer MTR beyond simple optimization of temperature and Mg:SiO_2_ ratio, by simultaneously considering both thermodynamic and kinetic aspects. Thermodynamically, the process window should be defined in the temperature–composition space where Si formation is favored over SiO_2_ and the formation of Mg silicate phases is suppressed. Kinetically, the reduction behavior is governed by the balance between Mg supply and surface reaction, which can be described using two competing descriptors: the Mg vapor impingement flux (J_input_) and the surface consumption term (k_reaction_(T)·N_s_), where N_s_ denotes the population of surface‐adsorbed Mg species. Previous studies have mainly focused on phase evolution as a function of temperature, Mg:SiO_2_ molar ratio, and pressure [20, 25, 26, 27], whereas supply‐consumption balance‐based process design for morphology‐controlled Si synthesis remains limited.

In this work, a thermo‐kinetic process model is proposed and used to regulate reaction conditions to maintain the supply‐consumption balance, enabling the conversion of Stöber SiO_2_ into monodisperse spherical porous Si without aggregation. In addition, to preserve this morphology during the subsequent purification step, a carbon exoskeleton strategy is introduced before acid etching so that the fragile porous framework can remain mechanically supported throughout the removal of MgO and residual silica. The resulting carbon‐coated monodisperse spherical porous Si is then applied as a Li‐ion battery anode and evaluated electrochemically, demonstrating that integrating thermo‐kinetic process design with a carbon exoskeleton strategy provides an effective process platform for producing uniform, monodisperse spherical porous Si.

## 2 Results and Discussion

Figure 1 schematically illustrates a process‐design framework for Mg‐vapor‐based MTR, in which the reaction behavior is discussed in terms of the balance between Mg supply to the precursor surface and surface consumption. As shown in Figure 1a, the SiO_2_ precursor bed and the Mg source were vertically separated to simplify the Mg vapor delivery pathway, allowing Mg vapor generated from the lower Mg bed to be introduced into the upper SiO_2_ layer. The SiO_2_ layer was supported on a perforated plate and a stainless‐steel mesh, which mitigated localized overheating and reduced the possibility of direct‐contact‐driven reaction non‐uniformity, while providing a controllable basis for regulating Mg supply.

**FIGURE 1:**
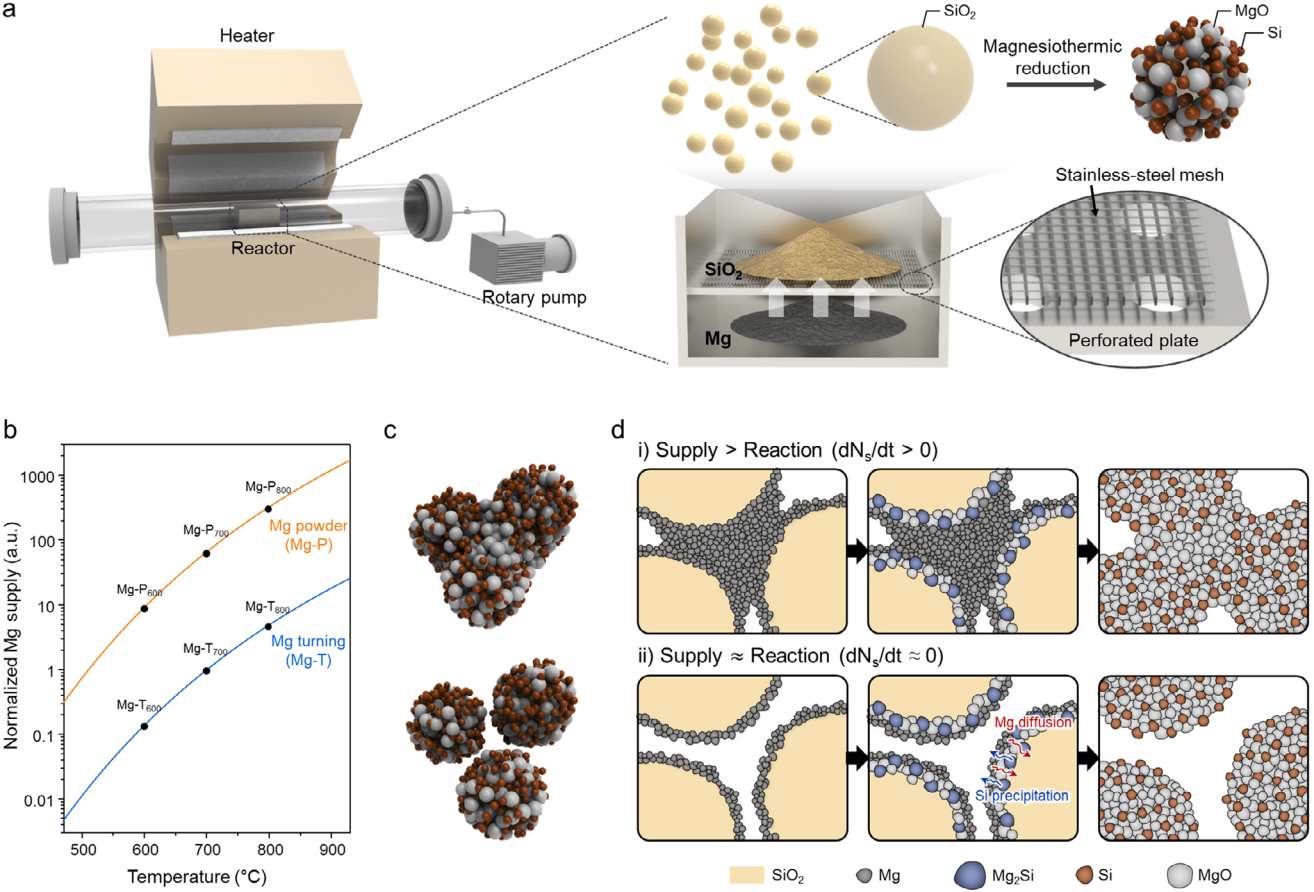
Process‐design framework for Mg vapor magnesiothermic reduction (MTR) governed by a supply‐consumption balance. (a) Schematic of the reactor configuration with a vertically separated Mg source and SiO_2_ precursor bed supported on a perforated plate and a stainless‐steel mesh. (b) Normalized Mg supply level as a function of temperature for Mg powder and Mg turnings, with the investigated conditions indicated. (c) Representative particle morphologies illustrating agglomerated (upper) and discrete spherical particles (lower). (d) Schematic reaction pathways under supply‐dominated (dN_s_/dt > 0) and balanced (dN_s_/dt ≈ 0) regimes.

To rationalize the process behavior, the surface coverage of adsorbed Mg species on the precursor surface, N_s_, was introduced as a descriptive state variable. The temporal evolution of N_s_ can be expressed as a balance between a supply term and a consumption term:

(1) $$\begin{array}{cccc} & \;\frac{dN_{s}}{dt}=J_{\mathrm{supply}}\;-k_{\mathrm{reaction}}\left(T\right)N_{s} & & \end{array}$$

where t is the reaction time, and N_s_ denotes the effective surface coverage of adsorbed Mg species. J_supply_ is the incident flux of Mg vapor reaching the precursor surface, which can be approximated by the impingement flux based on the kinetic theory of gases:

(2) $$\begin{array}{cccc} & J_{\mathrm{supply}}\approx \frac{P_{\mathrm{Mg}}}{\sqrt{2\pi m_{\mathrm{Mg}}k_{B}T}} & & \end{array}$$

where P_Mg_ is the local Mg partial pressure near the precursor surface, m_Mg_ is the mass of an Mg atom, k_B_ is the Boltzmann constant, and T is the reaction temperature.

The consumption term represents the depletion of adsorbed Mg species via the surface reduction reaction. For regime comparison, the consumption term was approximated as first order with respect to N_s_, i.e., k_reaction_(T)N_s_, and the effective rate constant was assumed to follow Arrhenius behavior:

(3) $$\begin{array}{cccc} & k_{\mathrm{reaction}}\left(T\right)=k_{0}\;\exp\left(-\frac{E_{a}}{k_{B}T}\right) & & \end{array}$$

where k_0_ is the pre‐exponential factor and E_a_ is the activation energy.

Figure 1b shows that the normalized Mg supply level varies with temperature depending on the Mg source morphology. Because the SiO_2_ precursor and the Mg source share the same furnace zone and thus the same temperature, the effective local Mg partial pressure near the precursor can still differ at an identical temperature as a function of source morphology, thereby shifting J_supply_. From a scaling perspective, the effective local P_Mg_ is expected to increase with both P_vap_(T) and A_source_ (defined at a fixed Mg mass), under otherwise comparable transport conditions. Accordingly, a larger A_source_ can contribute to a higher effective local P_Mg_ at a given temperature by providing a larger evaporation interface, leading to a higher J_supply_. To examine this effect, Mg powder (Mg‐P) and Mg turning (Mg‐T) were employed at an identical Mg mass, and their source‐dependent supply levels were compared. Under this condition, the two sources exhibited markedly different BET specific surface areas (2.7410 and 0.0415 m^2^ g^−1^ for Mg‐P and Mg‐T, respectively). (Figure S1) Together with morphology‐dependent evaporation behavior, this difference can shift the effective local P_Mg_ and thereby J_supply_ even at an identical temperature, consistent with the separated supply levels mapped in Figure 1b. On this basis, six process conditions were constructed at 600°C, 700°C, and 800°C using Mg‐P (Mg‐P_600_, P_700_, P_800_) and Mg‐T (Mg‐T_600_, T_700_, T_800_), and Figure 1b maps these conditions onto the supply‐level axis.

Figure 1c schematically illustrates how the relative magnitude of the supply and consumption terms can influence particle agglomeration. When J_supply_ exceeds k_reaction_(T)N_s_ such that dN_s_/dt > 0, excessive surface accumulation of Mg can promote localized growth at interparticle contact regions, increasing the likelihood of neck formation and interparticle bridging. When dN_s_/dt ≈ 0, the system approaches a quasi‐steady state, which can be favorable for preserving spherical morphology and maintaining a monodisperse structure. When dN_s_/dt < 0, Mg supply cannot keep pace with surface consumption, which can lead to incomplete reduction or residual SiO_2_ and magnesium silicate phases.

These behaviors are further illustrated in the microscopic reaction‐pathway schematic in Figure 1d. In both regimes, Mg diffuses through the pore network and is consumed at the precursor surface leading to Si formation [28]. Under a supply‐dominated condition, the amount of Mg accumulated on the surface can become significant relative to the consumption rate in the reduction reaction, enabling reaction products to grow along contact regions into a neck‐like morphology and thereby facilitating interparticle bridging. In contrast, under a balanced supply‐reaction condition, Mg supply and consumption proceed in a more comparable manner, limiting excessive Mg accumulation and favoring reaction pathways that suppress interparticle bridging while supporting spherical shape retention and monodispersity. This framework provides a consistent basis for comparing differences in phase composition, agglomeration, and morphological retention observed in subsequent results in terms of the supply‐reaction balance.

Figure 2 compares the phase composition and microstructural evolution across process conditions, thereby providing experimental validation of the supply‐consumption framework proposed in Figure 1. Figure 2a presents x‐ray diffraction (XRD) patterns of the reduced products as a function of Mg source morphology and reaction temperature. The overall MTR reaction can be explained as following Equation (4):

(4) $$\mathrm{Si}O_{2}+2\mathrm{Mg}\to \mathrm{Si}+2\mathrm{MgO}$$

**FIGURE 2:**
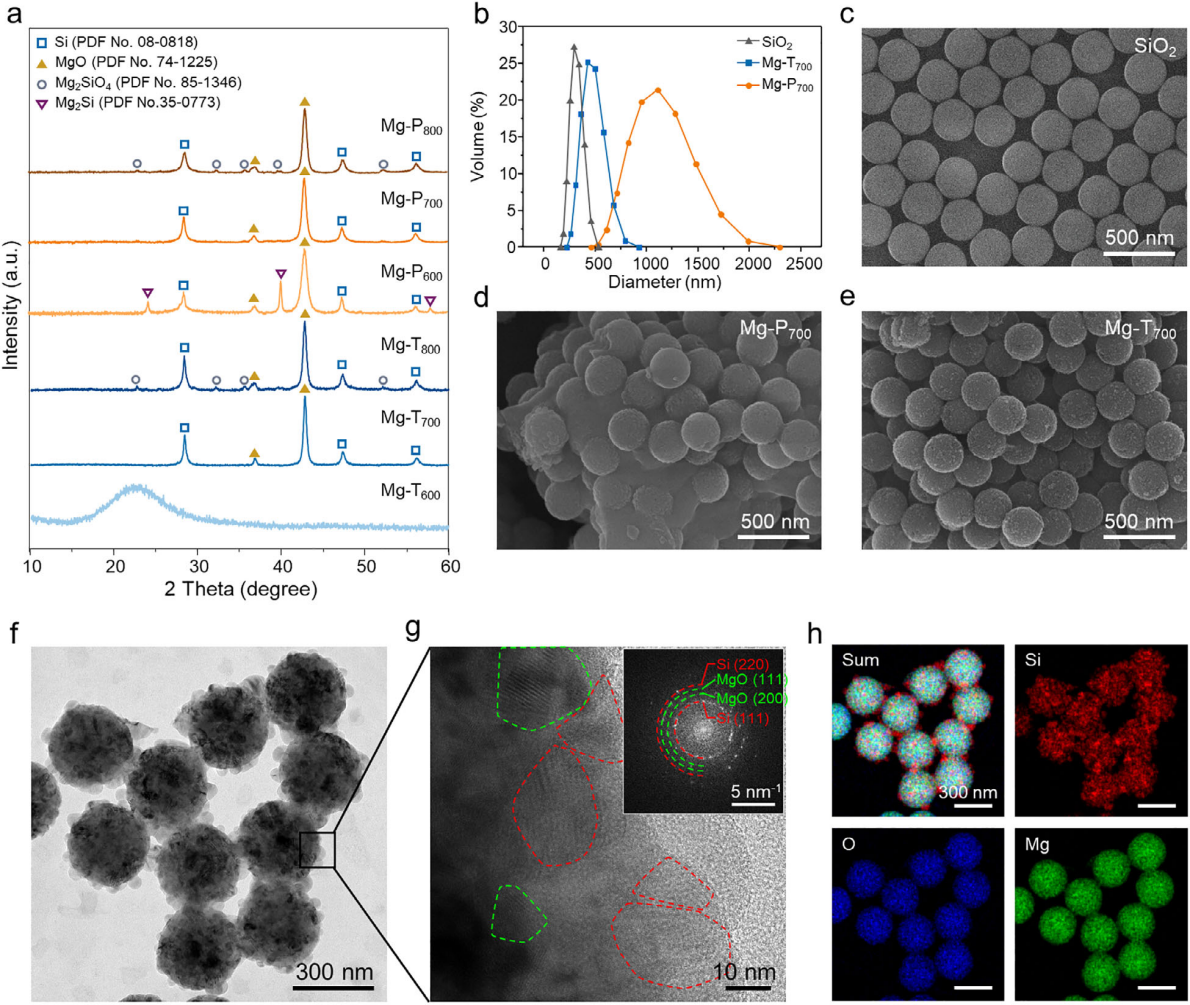
Phase and morphology characterization of MTR products obtained with different Mg sources and reaction temperatures. (a) XRD patterns of the reduced products obtained using different Mg sources and reaction temperatures (b) Particle size distributions of the SiO_2_ precursor and representative products, showing morphology‐dependent broadening associated with agglomeration. (c–e) SEM images comparing (c) the precursor, (d) Mg‐P_700_, and (e) Mg‐T_700._ (f) TEM image of the representative product exhibiting retained spherical morphology. (g) HRTEM image and corresponding FFT (inset) confirming the coexistence of crystalline Si and MgO domains. (h) STEM‐EDS elemental maps showing the spatial distributions of Si, O, and Mg.

In practice, the reaction is often described by a stepwise pathway involving an Mg_2_Si intermediate: [23, 28]

Reaction step 1:

(5) $$\mathrm{Si}O_{2}+4\mathrm{Mg}\to Mg_{2}\mathrm{Si}+2\mathrm{MgO}$$

Reaction step 2:

(6) $$Mg_{2}\mathrm{Si}+\mathrm{Si}O_{2}\to 2\mathrm{Si}+2\mathrm{MgO}$$

Depending on the conditions, Mg_2_SiO_4_ can also form when the MgO generated during reduction further reacts with residual SiO_2_ [24], as expressed by

(7) $$\mathrm{Si}O_{2}+2\mathrm{MgO}\to Mg_{2}\mathrm{Si}O_{4}$$

In Mg‐T_600_, a broad amorphous peak attributed to SiO_2_ was observed around 2θ = 23°, indicating incomplete reduction. This is consistent with a supply‐limited regime in which the turning source provides a relatively small A_source_ at 600°C, resulting in a lower local P_Mg_ near the precursor and, consequently, an insufficient J_supply_. In contrast, for the Mg‐P_600_, Mg_2_Si ‐related reflections were detected together with Si and MgO peaks. This indicates that a higher J_supply_, enabled by the larger A_source_ of the powder source, promotes progression along the Mg_2_Si formation pathway (Equation 5). However, at 600°C the solid‐state conversion of Mg_2_Si to Si via Equation (6) can be limited by reaction kinetics, so Mg_2_Si may remain as an intermediate phase [20]. At 800°C (Mg‐T_800_ and Mg‐P_800_), Mg_2_SiO_4_ peaks were detected in addition to Si and MgO, suggesting that the conversion of MgO into a silicate phase through further reaction with residual SiO_2_ is expected to be thermodynamically more favorable at elevated temperature (Equation 7) [29]. At 700°C, regardless of Mg source morphology, Si and MgO were dominant, and impurity phase signals were relatively suppressed, indicating that 700°C provides a temperature window for obtaining phase‐pure reduction products. Despite this similarity in phase composition, the final particle morphology and aggregation state remained strongly dependent on the Mg source. This difference is not readily explained by phase thermodynamics alone and is instead interpreted in terms of the supply‐reaction balance described in Figure 1.

Figure 2b compares the particle size distributions of the pristine SiO_2_ precursor, Mg‐T_700_, and Mg‐P_700_. The median particle diameter (D_50_) increased from 390 nm for SiO_2_ to 485 nm for Mg‐T_700_ while maintaining a relatively narrow distribution. In contrast, Mg‐P_700_ showed a substantially broadened distribution with D_50_ = 1.25 µm and a pronounced tail toward larger equivalent diameters. This suggests that the relatively higher J_supply_ in Mg‐P_700_ promotes Mg accumulation during the reaction, which is consistent with interparticle bridging and the resulting increase in apparent particle size. (Figure S2) Consistent with this interpretation, scanning electron microscopy (SEM) images (Figure 2c–e) showed pronounced agglomerate formation for Mg‐P_700_, whereas Mg‐T_700_ retained well‐separated spherical particles with superior morphology preservation. These results indicate that even when the phase composition at 700°C is similar, differences in J_supply_ governed by the effective surface area of the Mg source critically determine agglomeration and morphology retention.

The microstructure of the optimized Mg‐T_700_ product was further examined by Transmission electron microscopy (TEM) and scanning transmission electron microscopy coupled with energy‐dispersive x‐ray spectroscopy (STEM‐EDS). In Figure 2f, the Mg‐T_700_ product retained a spherical morphology while forming an internal nanocomposite structure. High‐resolution TEM (HRTEM) and fast Fourier transform (FFT) analyses showed d‐spacings corresponding to characteristic lattice planes of both Si and MgO, confirming the coexistence of nanocrystalline domains. (Figure 2g) STEM‐EDS mapping also showed Mg, O, and Si signals distributed throughout the particle, supporting the formation of an Si/MgO nanocomposite. (Figure 2h) These results show that the reaction behavior varied markedly with process conditions, as evidenced by incomplete reduction under insufficient Mg supply, the presence of Mg_2_Si intermediates at low temperature, agglomeration under excessive Mg supply, and silicate formation at high temperature. Among the conditions examined, Mg‐T_700_ most effectively satisfied the dual requirement of phase purity and morphology retention.

Figure 3 illustrates the role of a carbon exoskeleton strategy for preserving particle integrity during post‐etching and identifies an appropriate coating window for forming a mechanically supportive carbon shell. After MTR, the product formed as a Si/MgO nanocomposite, and subsequent acid etching to remove MgO was required to obtain Si. Based on the overall reaction (Equation 1), MgO can account for approximately 74 wt.% (approximately 65 vol%) of the composite; therefore, removing MgO generates substantial internal void space and renders the remaining framework mechanically fragile. Accordingly, an external reinforcement strategy is required to suppress particle collapse during etching, washing, and drying while retaining the spherical morphology.

**FIGURE 3:**
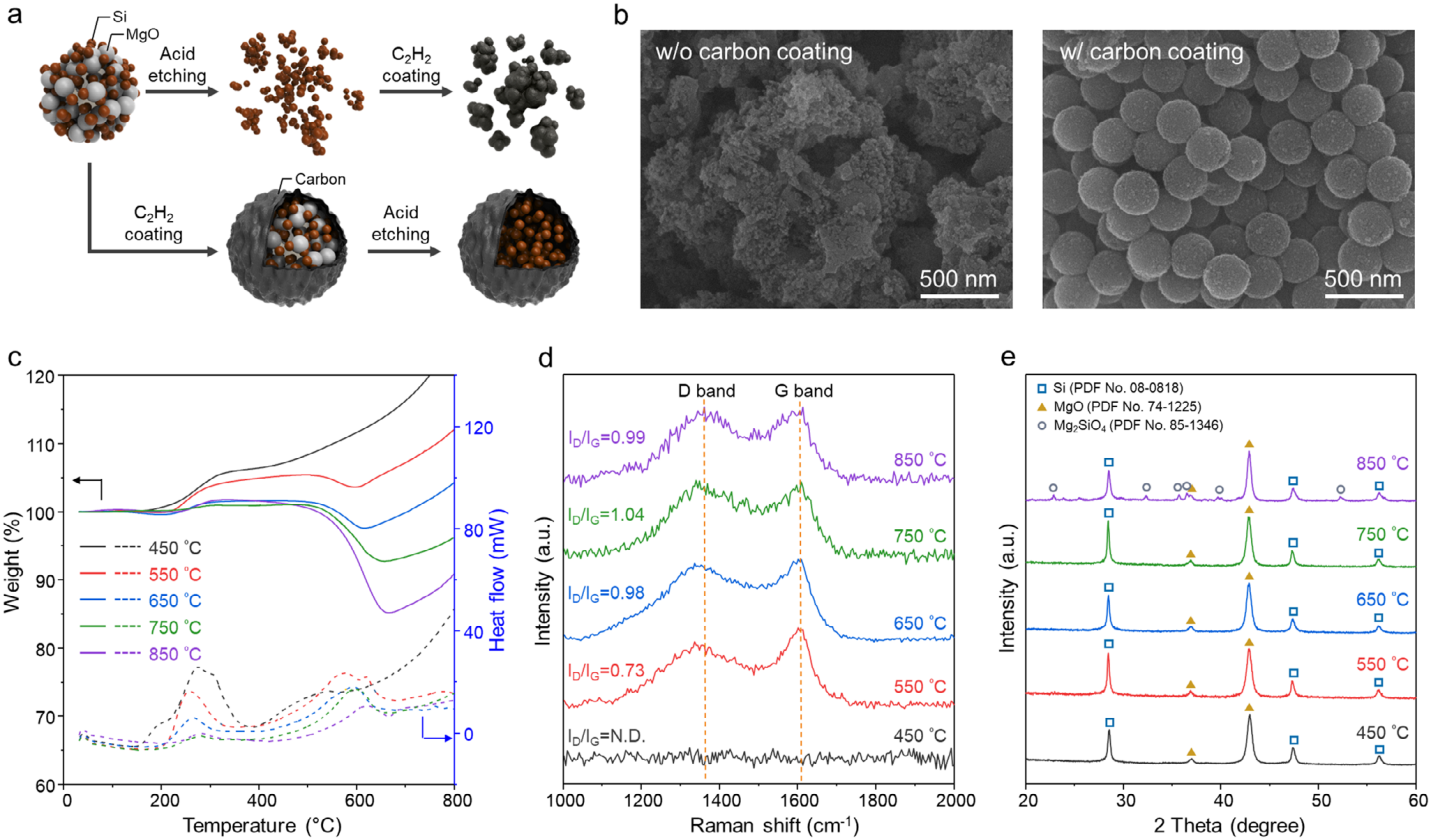
Carbon‐exoskeleton strategy and optimization of coating conditions for morphology preservation during post‐etching. (a) Schematic illustration of the processing routes comparing etching‐first and carbon‐precoating approaches. (b) SEM images of etched products prepared without and with carbon coating. (c) TGA‐DSC profiles measured in air for carbon‐coated samples prepared at 450°C, 550°C, 650°C, 750°C, and 850°C. (d) Raman spectra of the carbon‐coated samples, with the corresponding I_D_/I_G_ values. (e) XRD patterns after carbon coating at different temperatures to evaluate phase stability during the coating step.

To address this issue, a carbon exoskeleton was introduced by depositing a carbon layer prior to acid treatment so that the particle exterior remains mechanically supported during and after etching. Figure 3a schematically depicts that, whereas etching the Si/MgO composite prior to reinforcement can weaken the porous framework and trigger morphological collapse, precoating with carbon enables the outer shell to remain mechanically supportive during the subsequent removal of MgO and residual silica, thereby retaining spherical shape and particle dispersibility after acid treatment. Figure 3b shows the corresponding SEM images. Severe morphological collapse was observed after acid leaching without carbon coating, whereas carbon‐coated particles retained a discrete spherical morphology.

Carbon coating was performed under an acetylene (C_2_H_2_) atmosphere at 450°C, 550°C, 650°C, 750°C, and 850°C. Figure 3c shows thermogravimetric analysis‐differential scanning calorimetry (TGA‐DSC) profiles acquired under an oxidative atmosphere, demonstrating that the oxidation resistance and carbon‐related combustion behavior depend strongly on the coating temperature. For the sample coated at 450°C, a larger mass gain attributable to Si oxidation was observed starting near 200°C [30], whereas increasing the coating temperature led to a reduced extent of Si oxidation, consistent with the formation of a protective carbon layer [31]. The temperature‐dependent mass‐change behavior can be understood by considering the partial overlap between Si oxidation and carbon combustion. The mass loss observed around 500°C–650°C, accompanied by an exothermic DSC feature, can be attributed mainly to combustion of the carbon component [32]. Although Si oxidation may also occur concurrently in this temperature range, the net mass loss accompanied by the exothermic DSC feature indicates that carbon combustion dominates the net mass change. Above approximately 650°C, as the carbon‐combustion‐related mass‐loss contribution becomes much less pronounced, the net mass change is governed mainly by ongoing Si oxidation, resulting in the observed mass gain. Because Si oxidation and carbon combustion partially overlap, the absolute carbon content cannot be quantified unambiguously based solely on the mass loss in a specific temperature range. Therefore, the mass‐change values obtained from the assigned temperature regions are provided in Table S1 for a comparative evaluation of the coating‐temperature‐dependent oxidation and carbon‐combustion behavior.

Figure 3d shows Raman spectra of the coated samples. No carbon‐related signal was observed at 450°C, whereas clear D and G bands appeared above 550°C at approximately 1350 and 1600 cm^−1^, respectively, indicating that carbon formation became significant above this temperature. For acetylene‐derived disordered carbon, the temperature‐dependent I_D_/I_G_ variation reflects structural evolution rather than a simple monotonic change in the degree of graphitization. For disordered carbon, I_D_/I_G_ is affected by the formation of sp^2^ carbon domains, defect density, domain size, and nanocrystalline ordering [29]. As the coating temperature increased from 550°C to 650°C, progressive dehydrogenation and the formation of defect‐rich sp^2^ carbon domains promoted the development of sp^2^ network, thereby increasing the disorder‐related D band contribution relative to the G band. At 750°C–850°C, structural rearrangement and improved ordering at higher temperature can reduce I_D_/I_G_ [33, 34]. Powder electrical measurements showed that the inverse resistance increased from 8.82 × 10^−6^ Ω^−1^ at 550°C to 3.38 × 10^−1^ Ω^−1^ at 850°C, indicating improved electrical connectivity of the carbon‐coated powder with increasing coating temperature. (Table S2) The behavior is consistent with improved electrical connectivity of the carbon coating formed at higher temperature. Although carbon features appeared above 550°C, the mere presence of carbon was not sufficient to ensure morphology retention during post‐etching processing. For samples coated at 550°C and 650°C, partial particle collapse was observed after acid etching and washing, suggesting that the exoskeleton may not have been sufficiently continuous to provide effective mechanical support. (Figure S3) In contrast, the carbon exoskeleton formed at 750°C preserved both spherical morphology and particle dispersibility after etching.

Phase stability during carbon coating was evaluated by XRD. (Figure 3e) While Si and MgO peaks were retained, additional Mg silicate reflections were observed at coating temperatures of 850°C and above, indicating that the coating temperature must be selected within a process window that promotes carbon formation while suppressing thermally induced side reactions and phase transformation. Taken together, these results identify 750°C as the most appropriate coating temperature for simultaneously achieving carbon formation, morphology retention, and phase stability.

Figure 4 presents the pore structure, phase purity, and structural integrity of the final product obtained after HCl etching followed by an additional HF etching step to remove residual SiO_2_ and obtain a purified Si@C product. Brunauer‐Emmett‐Teller (BET) analysis showed that the specific surface area increased markedly from 28.24 m^2^ g^−1^ before etching to 235.22 m^2^ g^−1^ after etching. (Figure 4a) In addition, the Barrett–Joyner–Halenda (BJH) pore volume increased from 0.099 to 0.833 cm^3^ g^−1^. (Table S3) These increases indicate the development of an internal pore network as MgO and residual SiO_2_ are removed during the acid treatments. The final porous Si@C product showed a tap density of 0.41 g cm^−3^, comparable to those of reported porous secondary‐particle Si‐based anodes, suggesting that the retained microspherical morphology helps maintain powder packing even after the formation of internal porosity. (Figure S41) [35, 36, 37, 38].

**FIGURE 4:**
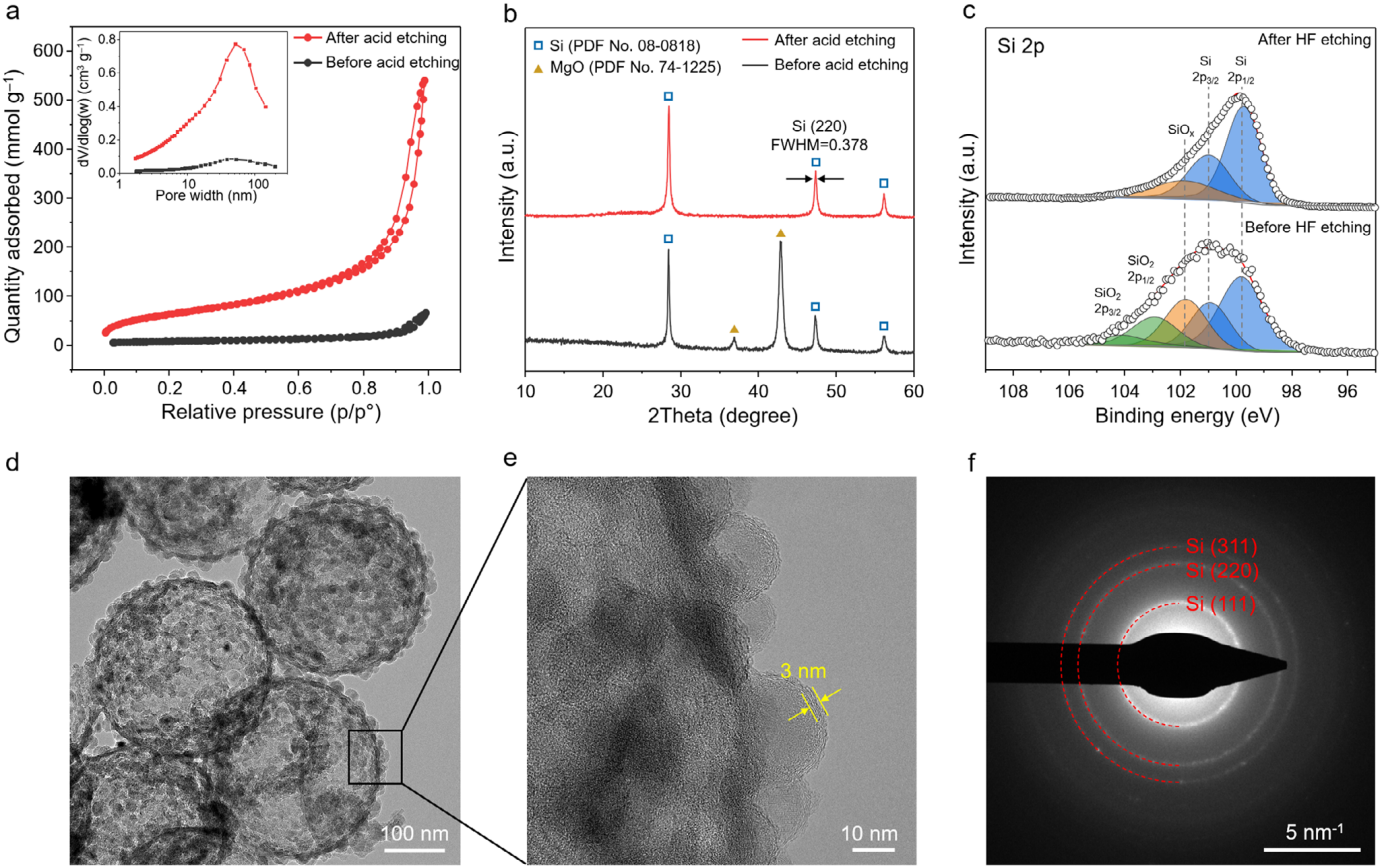
Structural and compositional evolution upon acid etching and characterization of the final Si@C product. (a) N_2_ adsorption–desorption isotherms, with the corresponding pore‐size distribution (inset). (b) XRD patterns showing phase evolution upon acid etching. (c) Si 2p XPS spectra showing the removal of residual SiO_2_‐related species after the additional acid etching step. (d) TEM image of the etched product showing retained spherical morphology with an internal porous network. (e) High‐magnification TEM image confirming a continuous carbon layer of approximately 3 nm on the particle surface. (f) SAED pattern with diffraction rings consistent with crystalline Si.

The phase evolution during acid etching was examined by XRD, as shown in Figure 4b. Prior to etching, diffraction peaks from MgO coexisted with the Si reflections, whereas after etching the MgO peaks were no longer observed and the product was dominated by the Si phase. The crystallite size was estimated from the Si (220) reflection at 2θ = 47.3° using the measured peak broadening (FWHM = 0.378°) and the Scherrer equation, yielding a crystallite size of 23.8 nm. Figure 4c compares the Si 2p XPS spectra after HCl etching and after the subsequent HF etching step. Before HF etching, SiO_2_^−^ and SiO_x_‐related components were observed, indicating the presence of residual oxidized Si species that were not completely reduced during MTR. After HF etching, the SiO_2_‐related components were largely removed, while the elemental Si 2p_3/2_ and Si 2p_1/2_ peaks became dominant, confirming the effective removal of residual SiO_2_ from the product. The apparent Si‐equivalent content of the final Si@C product was further estimated from the final residual mass after oxidative TGA. Assuming complete combustion of carbon and complete oxidation of Si‐containing species to SiO_2_ at 1300°C, the final residual mass of 143.4% corresponds to an apparent Si‐equivalent content of approximately 67.0 wt.%. (Figure S5)

TEM images showed that the spherical particle morphology was retained after etching, while a porous network developed throughout the particle interior. (Figure 4d) A magnified TEM image further confirmed the presence of a continuous carbon layer of approximately 3 nm on the particle surface. (Figure 4e) The selected area electron diffraction (SAED) pattern shown in Figure 4f exhibited diffraction rings assignable to Si (111), (220), and (311), corroborating that the final product consists of crystalline Si.

Figure 4 confirmed that acid etching yielded phase‐pure Si while preserving the particle morphology supported by the carbon exoskeleton and generating an internal pore network. Accordingly, Figure 5 evaluates how the associated structural characteristics correlated with electrochemical behavior by comparing three electrodes. Mg‐T_700_ exhibited discrete spherical porous particles, whereas Mg‐P_700_ showed agglomerated products formed under an Mg‐oversupply condition. In addition, to examine the role of porosity independently, Mg‐T_700_‐RP (reduced porosity) was prepared by vacuum drying and compression, thereby promoting closer contact between the carbon framework and Si and partially reduced the pore structure. (Figure S6) The effects of particle agglomeration and porosity on cell performance were compared under identical test conditions.

**FIGURE 5:**
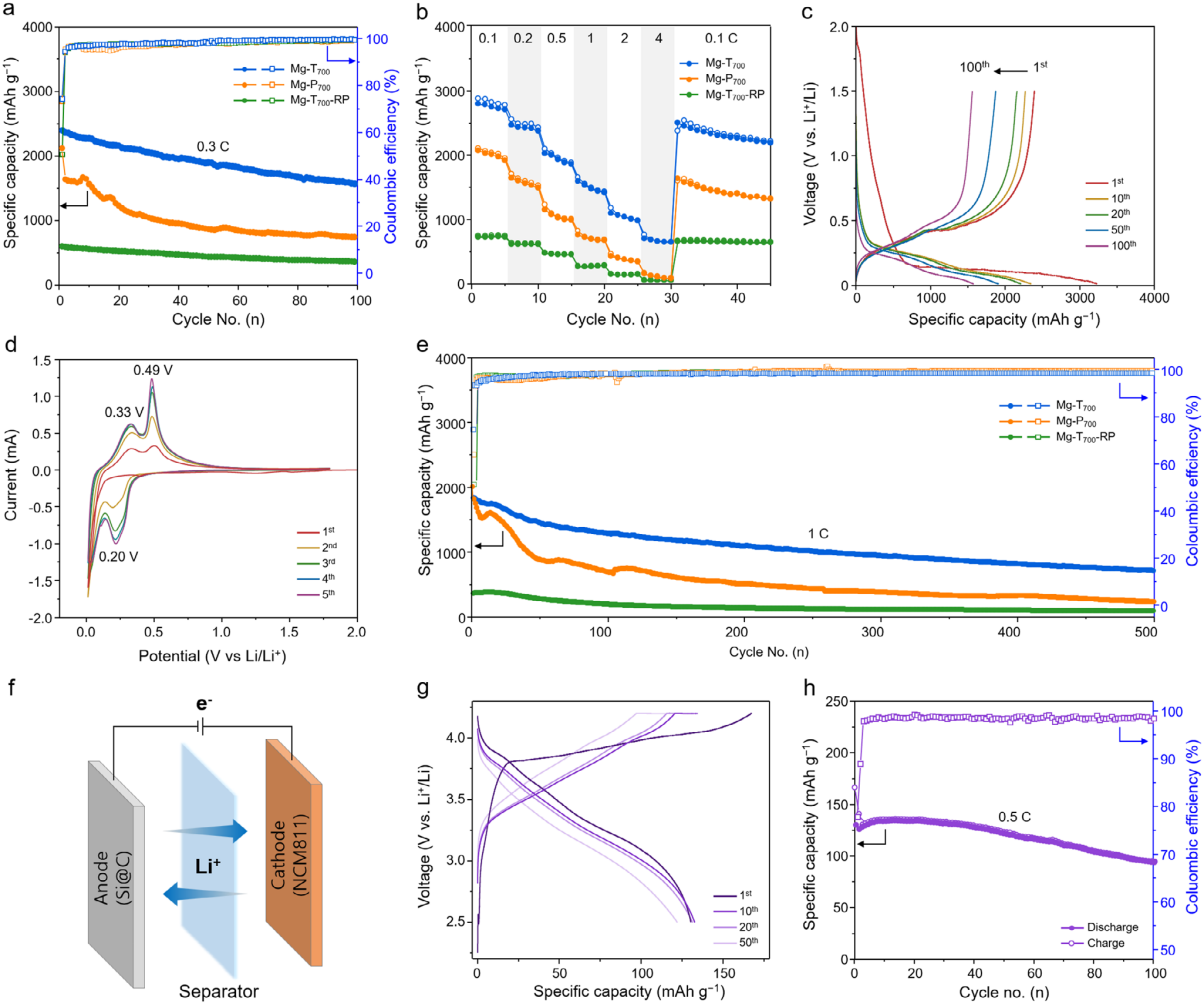
Electrochemical performance of Si@C anodes prepared with different Mg sources and post‐treatments. (a) Half‐cell cycling performance at 0.3 C, showing specific capacity (left axis) and coulombic efficiency (right axis) for Mg‐T_700_, Mg‐P_700_, and Mg‐T_700_‐RP. (b) Rate capability evaluated under stepwise C‐rate conditions. (c) Selected‐cycle galvanostatic charge–discharge profiles of Mg‐T_700_. (d) Cycle‐dependent CV curves of Mg‐T_700_. (e) Long‐term cycling performance measured at 1 C. (f) Schematic illustration of the Si@C//NCM811 full‐cell configuration. (g) Charge‐discharge voltage profiles of the Mg‐T_700_‐based full cell at selected cycles. (h) Cycling performance of the Mg‐T_700_‐based full cell.

Figure 5a compares the half‐cell cycling performance measured at 0.3 C. Mg‐T_700_ delivered the highest reversible capacity and the most gradual capacity decay among the three samples, while maintaining a high coulombic efficiency after the initial cycle. The initial discharge and charge capacities were 3225.2 and 2392.6 mAh g^−1^, respectively, with an initial coulombic efficiency of 74.2%. Even after prolonged cycling, a reversible capacity of 1571.1 mAh g^−1^ was retained at the 100th cycle. In contrast, Mg‐P_700_ exhibited a relatively high initial capacity and a comparable initial coulombic efficiency of 73.2%, but its capacity faded more rapidly upon cycling, consistent with less uniform electrolyte access and more heterogeneous reaction and transport behavior within the agglomerated particle assembly. Mg‐T_700_‐RP showed a relatively low reversible capacity from the first cycle, along with a low initial coulombic efficiency of 50.6%, and maintained a generally lower capacity throughout cycling. This suggests that pore suppression restricts the electrochemically accessible Si domains and internal Li‐ion transport pathways.

The rate capability results in Figure 5b are consistent with this trend. Mg‐T_700_ maintained a relatively high capacity even under stepwise increases in current density and showed a capacity recovery of 80.1% upon returning to the low‐rate condition. By contrast, Mg‐P_700_ exhibited a larger capacity loss at high rates, which can be attributed to non‐uniform local current distribution and ion/electron transport arising from the agglomerated morphology. Mg‐T_700_‐RP maintained lower capacities over the entire C‐rate range, and the performance deterioration became more pronounced at higher rates. This behavior indicates aggravated kinetic limitation due to restricted electrolyte penetration and Li‐ion transport caused by reduced porosity.

To examine the reaction evolution of Mg‐T_700_, selected‐cycle galvanostatic charge–discharge profiles and cyclic voltammetry (CV) behavior were compared in Figure 5c,d. The voltage profiles of Mg‐T_700_ retained a similar shape after the initial cycles, but gradual capacity decay became evident as cycling proceeded. This behavior may be attributed to the progressive reduction in the electrochemically accessible Si fraction caused by repeated volume change, together with the accumulation of interfacial side reactions [39, 40, 41]. In the CV curves shown in Figure 5d, the cathodic peak near 0.20 V is associated with the formation of a lithiated Si phase upon Li insertion, whereas the anodic peaks near 0.33 and 0.49 V are related to the dealloying of Li_x_Si through Li extraction from the lithiated Si phase [42, 43]. In the CV results of Mg‐T_700_, these major redox peaks associated with Si lithiation/delithiation remained relatively distinct and reproducible over repeated cycles. In contrast, the cycle‐dependent CV curves of Mg‐P_700_ and Mg‐T_700_‐RP exhibited more pronounced peak broadening or intensity decay (Figure S7), indicating that the redox features of Mg‐T_700_ were retained with comparatively higher reproducibility over repeated cycles.

Figure 5e shows the long‐term cycling performance measured at 1 C. Mg‐T_700_ exhibited the most stable cycling durability even under the high current density condition and retained a capacity of 718.3 mAh g^−1^ after 500 cycles. In contrast, Mg‐P_700_ and Mg‐T_700_‐RP showed larger capacity decay, which can be attributed to structural limitations originating from agglomeration and reduced porosity, respectively. These results indicate that a well‐dispersed porous spherical architecture is advantageous not only in terms of initial capacity, but also in terms of sustained reaction accessibility and interfacial stability. For contextual comparison, representative porous Si‐based anodes reported in the literature are summarized in Table S4. Mg‐T_700_ shows competitive cycling performance while maintaining the morphology‐preserving design enabled by the controlled MTR process.

To verify the practical applicability of the optimized Mg‐T_700_‐derived Si@C electrode, a full cell was assembled using the Si@C anode and an NCM811 cathode, as shown in Figure 5f. The full‐cell charge–discharge profiles in Figure 5g were maintained without substantial distortion during repeated cycling, and the full cell in Figure 5h exhibited an initial reversible capacity of 130.2 mAh g^−1^ and an initial Coulombic efficiency of 77.8%. After 100 cycles, a capacity retention of 72.6% was maintained. These results support that the monodisperse spherical porous Si@C structure designed in this study can provide applicability not only at the half‐cell level but also in a full‐cell configuration.

Figure 6 presents a comprehensive set of electrochemical diagnostics to elucidate the electrochemical kinetic origin of the performance differences observed in Figure 5. In the pre‐cycling EIS results shown in Figure 6a, Mg‐P_700_ exhibited the largest semicircle, indicating that interfacial reaction and charge transfer were least favorable in the agglomerated particle assembly. In contrast, Mg‐T_700_ showed a relatively small semicircle, suggesting that a more favorable interfacial reaction environment was established by the well‐dispersed porous structure. Mg‐T_700_‐RP exhibited a semicircle size comparable to that of Mg‐T_700_ but showed a distinct response in the low‐frequency region. This behavior can be interpreted as reflecting restricted electrolyte accessibility and ion transport pathways caused by reduced porosity.

**FIGURE 6:**
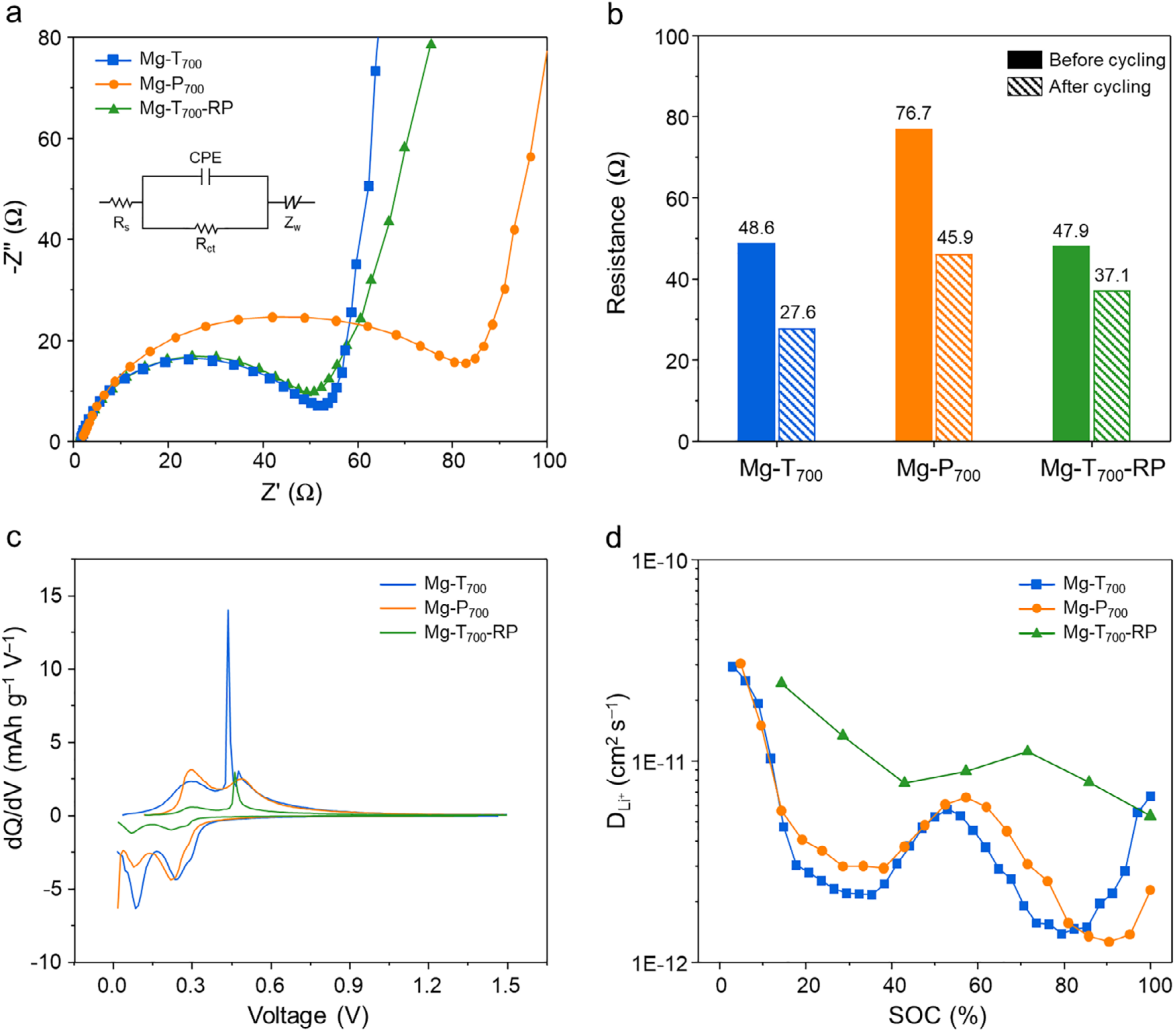
Electrochemical diagnostics for elucidating the kinetic origin of the performance differences among the three electrodes. (a) Pre‐cycling EIS Nyquist plots of the assembled cells. (b) Resistance summary derived from EIS analysis before and after cycling. (c) Normalized dQ/dV curves at the 10th cycle. (d) SOC‐dependent apparent Li^+^ diffusion coefficients derived from GITT during lithiation.

The resistance summary in Figure 6b quantitatively supports this trend. Mg‐P_700_ exhibited the largest resistance both before and after cycling, indicating that the agglomerated structure imposed the greatest electrode‐level interfacial/polarization penalty (Figure S8). Mg‐T_700_ maintained the lowest overall resistance, corresponding to the most favorable interfacial kinetics. Meanwhile, Mg‐T_700_‐RP showed an intermediate resistance behavior, higher than that of Mg‐T_700_ but lower than that of Mg‐P_700_. This indicates that pore suppression introduced a measurable kinetic penalty, but one that differs from the severe resistance increase caused by agglomeration in Mg‐P_700_ and is more closely associated with altered low‐frequency transport behavior.

Figure 6c compares the normalized dQ/dV curves at the 10th cycle. Mg‐T_700_ exhibited relatively sharp and well‐resolved redox features, indicating a more reproducible electrochemical reaction response. In contrast, Mg‐P_700_ showed more pronounced peak broadening and peak position shifts, suggesting a more heterogeneous reaction environment and increased polarization within the agglomerated structure. Mg‐T_700_‐RP displayed a peak distribution and feature shape distinct from those of Mg‐T_700_ and Mg‐P_700_, which can be interpreted as behavior consistent with reduced reaction accessibility caused by reduced porosity. This tendency was qualitatively consistent with the cycle‐dependent dQ/dV evolution shown in Figure S9. To further assess the charge‐storage kinetics associated with these structural differences, scan‐rate‐dependent CV analysis was performed. (Figure S10) Mg‐T_700_ and Mg‐P_700_ exhibited comparable b‐values of 0.71 and 0.69, respectively, indicating mixed diffusion‐ and surface‐controlled reaction behavior. In contrast, Mg‐T_700_‐RP showed a lower b‐value of 0.57, suggesting a stronger diffusion‐controlled contribution in the reduced‐porosity electrode. This result is consistent with the restricted electrolyte accessibility and internal Li‐ion transport pathways of Mg‐T_700_‐RP.

These differences were also consistently reflected in the SOC‐dependent apparent Li‐ion diffusion behavior derived from GITT. As shown in Figure 6d, all three samples exhibited relatively high apparent diffusion coefficients in the initial SOC region but followed different evolution profiles as lithiation proceeded. Mg‐T_700_ and Mg‐P_700_ showed a similar tendency in which the diffusion response decreased in the intermediate SOC region and then changed again, whereas Mg‐T_700_‐RP exhibited a clearly distinct diffusion evolution behavior over the entire SOC range. In the case of Mg‐T_700_‐RP, reduced porosity limited electrolyte penetration and internal transport pathways, so that lithiation proceeded through less accessible transport routes within the electrode structure. As a result, the apparent diffusion behavior also appeared as a profile distinct from those of the other two samples.

Overall, Figure 6 demonstrates that the performance differences among the three samples were not governed by a single common origin, but rather by different kinetic penalties. In Mg‐P_700_, agglomeration increased the interfacial resistance and reaction heterogeneity, which acted as the primary limiting factor. In contrast, Mg‐T_700_‐RP was more strongly affected by diffusion‐related limitation, as reduced porosity restricted the electrochemically accessible pathways and Li‐ion transport channels. By comparison, Mg‐T_700_ exhibited the most balanced behavior in terms of interfacial reaction, internal transport, and reaction distribution owing to its well‐dispersed porous spherical structure. This balanced kinetic behavior is consistent with the superior capacity retention, rate capability, long‐term stability, and full‐cell feasibility shown in Figure 5.

This balanced kinetic behavior is also reflected in the cross‐sectional electrode morphology after cycling. Cross‐sectional SEM images before and after 100 charge–discharge cycles show that the Mg‐T_700_ electrode exhibits a relatively moderate thickness increase from 16.1 to 22.7 µm, corresponding to ∼141% of its initial thickness. (Figure S11) In contrast, the Mg‐P_700_ electrode shows a much larger thickness increase from 15.4 to 30.8 µm (∼200%), consistent with more severe electrode swelling associated with the agglomerated particle assembly. The Mg‐T_700_‐RP electrode also increases from 14.7 to 23.3 µm (∼158%), indicating that the reduced‐porosity architecture provides limited structural tolerance against repeated volume changes. In contrast, Mg‐T_700_‐RP shows partially damaged particles, consistent with the limited structural tolerance of the reduced‐porosity architecture. In addition, post‐mortem TEM analysis (Figure S12) confirms that the cycled Si@C electrode still preserves an identifiable particle framework with a residual carbon layer and crystalline Si domains. These results are consistent with the improved structural retention of the optimized Mg‐T_700_ electrode during repeated cycling.

## 3 Conclusion

This study established an Mg‐vapor‐based magnesiothermic reduction strategy from a supply‐consumption perspective, showing that variations in effective Mg supply arising from source morphology and temperature lead to distinct phase and morphology outcomes, including differences in agglomeration and shape retention. To address the structural fragility of the Si/MgO composite during purification, a carbon exoskeleton was introduced prior to acid treatment, enabling morphology retention through sequential etching and yielding porous, phase‐pure Si. Electrochemical comparisons among a dispersed porous electrode, an agglomerated counterpart, and a reduced‐porosity variant further revealed that aggregation and porosity influence reversibility, rate capability, cycling stability, and kinetic behavior in different ways. Together, these results show that combining supply‐consumption‐controlled magnesiothermic reduction with carbon‐assisted morphology stabilization provides an effective route for producing morphology‐retaining porous Si@C anodes.

## 4 Experimental Section

### 4.1 Preparation of Si/MgO Composite Spheres

Magnesiothermic reduction of SiO_2_ was carried out at a fixed SiO_2_:Mg molar ratio while varying the reaction temperature and the size of the Mg source to probe the influence of the Mg supply rate. Spherical silica powder, 2.0 g (SiO_2_, Sukgyung AT Co., Ltd.), and magnesium, 1.78 g were charged into a stainless‐steel reactor and arranged in a vertically stacked two‐layer configuration with Mg at the bottom and SiO_2_ on top. Magnesium was introduced either as large turnings (Mg‐T, > 99%, Daejung) or as fine powder (Mg‐P, > 98%, Hana AMT), while keeping the SiO_2_:Mg molar ratio constant in all experiments. After loading, the reactor was evacuated to a pressure below 0.1 Torr, heated to selected temperatures in the range of 600°C–800°C at a ramp rate of 10°C min^−1^, and held at the target temperature for 3 h. After completion of the magnesiothermic reaction, the furnace was allowed to cool naturally to room temperature, and the reduced powders were collected.

### 4.2 Preparation of Carbon‐Coated Si (Si@C) Spheres

The reduced powders were placed in an alumina boat, which was transferred to a tube furnace operated at atmospheric pressure for carbon coating. The samples were heated under a flow of high‐purity Ar (99.999%, 200 sccm) to 450°C, 550°C, 650°C, 750°C, or 850°C at a ramp rate of 10°C min^−1^. Once the target temperature was reached, a gas mixture of C_2_H_2_ (20 sccm) and Ar (180 sccm) was introduced for 20 min to deposit a carbon shell. The furnace was then cooled to room temperature under flowing Ar.

The carbon‐coated powders were leached with 2 M hydrochloric acid (HCl, 35%–37%, Daejung) to remove residual Mg and Mg‐containing by‐products, followed by repeated washing with deionized water and ethanol until the supernatant reached neutral pH. Residual silica was removed by additional etching in 5 wt.% aqueous hydrofluoric acid solution (HF, prepared from 49% HF, Samchun), and the same washing procedure was applied until the supernatant again reached neutral pH. After etching, the C@Si samples were dried in a convection oven at 80°C overnight. In contrast, the reduced‐porosity sample was prepared using the same procedures, except that the final drying step was carried out in a vacuum oven at 80°C.

### 4.3 Characterization

The morphology of the samples was examined by field‐emission scanning electron microscopy (FE‐SEM, S‐4300SE, Hitachi). Transmission electron microscopy (TEM, Talos F200X G_2_, Thermo Fisher Scientific) operated at 200 kV and equipped with an energy‐dispersive x‐ray spectroscopy (EDS) detector was used to probe the internal structure, local crystallinity, and elemental distribution of individual particles. Crystal structure and phase composition were characterized by x‐ray diffraction (XRD, D8 Advance, Bruker) using Cu Kα radiation (λ = 0.154 nm). Surface chemical composition and valence states were analyzed by x‐ray photoelectron spectroscopy (XPS, VersaProbe PHI 5000, ULVAC‐PHI); the binding‐energy scale was referenced to the C 1s peak at 284.8 eV. Raman spectra were collected on a UHTS‐300 spectrometer (WITec) using a 532 nm excitation laser to confirm the presence and structural features of the carbon species. Thermogravimetric/differential scanning calorimetry analysis (TGA/DSC, STA7300, Hitachi) was performed in air from room temperature to 1300°C at a heating rate of 10°C min^−1^ to evaluate the carbon‐combustion and Si‐oxidation behavior. The apparent Si‐equivalent content of the final Si@C product was estimated from the final residual mass, assuming complete combustion of carbon and complete oxidation of Si‐containing species to SiO_2_. Nitrogen adsorption–desorption isotherms were measured using a Micromeritics TriStar II 3020 analyzer to determine the specific surface area and textural properties, including pore characteristics. The electrical resistance of the powder samples was measured using a four‐terminal method with a HIOKI RM3544 resistance meter. For each measurement, 0.5 g of powder was loaded into the measurement cell and compressed under a pressure of 20 MPa.

### 4.4 Electrochemical Measurements

#### 4.4.1 Half‐Cell

Electrodes were fabricated from a slurry containing Si@C spheres (active material), Super P (conductive carbon), and polyacrylic acid (PAA, 2 wt.% aqueous solution) at a mass ratio of 80:10:10. The components were mixed in a planetary paste mixer (THINKY, AR‐100) until a homogeneous dispersion was obtained. The slurry was cast onto 18 µm‐thick Cu foil using a doctor‐blade coater, dried in air at room temperature for 1 h, and further dried in a vacuum oven at 50°C overnight. Circular electrodes with a diameter of 12 mm were then punched from the dried films, yielding an active‐material loading of 1 ± 0.1 mg cm^−2^.

Electrochemical performance was evaluated in CR2032 coin‐type half cells assembled in an Ar‐filled glove box with the H_2_O concentration maintained below 0.01 ppm. Lithium metal was used as both counter and reference electrode, and a polyethylene (PE) separator was employed. The electrolyte was 1.3 M LiPF_6_ in an ethylene carbonate/diethyl carbonate mixture (EC/DEC, 1:1 w/w) containing 10 vol% fluoroethylene carbonate (FEC). Galvanostatic charge–discharge tests were conducted using an automatic battery cycler (WBCS3000, WonATech).

Before the main measurements, the cells underwent an activation protocol consisting of a formation cycle at 0.05 C. For capacity evaluation, the cells were subsequently cycled at 0.3 C between 0.01 and 1.5 V (vs. Li/Li^+^). Cyclic voltammetry (CV) and electrochemical impedance spectroscopy (EIS) were performed on a potentiostat (VSP, Bio‐Logic). CV curves were recorded in the potential range of 0.01–1.8 V (vs. Li/Li^+^) at scan rates of 0.1–1.0 mV s^−1^ to probe the initial redox reactions, solid–electrolyte interphase formation, and charge‐storage mechanism.

#### 4.4.2 Full‐Cell

The full cells were assembled using a high‐nickel NCM cathode material (N86, POSCO Future M). The cathode electrode was prepared by mixing NCM, polyvinylidene fluoride (PVDF) as a binder in N‐methyl‐2‐pyrrolidone (NMP) and the conducting agent (Super P) at a weight ratio of 90:5:5. The obtained slurry was coated onto a 20 µm‐thick Al foil using a doctor blade coater. The N/P ratio was set to 1.1 based on a practical reversible capacity of 2000 mAh g^−1^ for the Si@C anode.

For the assembled Si@C//NCM full‐cell, the cell was charged with a constant current (CC) at 0.05 C to 4.2 V, followed by a constant voltage (CV) step until the current decreased to 0.05 C before being discharged to 2.5 V with a constant current at 0.05 C. From the second cycle, the cell was cycled in the constant‐current (CC) mode within 2.5 and 4.2 V at a 0.5 C rate.

## Author Contributions

**Xiaojie Zhang**: data curation, formal analysis. **Xue Dong**: formal analysis. **Hak Ki Yu**: conceptualization, supervision, writing – original draft, writing – review and editing. **Junyoung Mun**: conceptualization, writing – review and editing, supervision. **Byung Joo Jeong**: data curation. **Hyeon‐Seok Bang**: formal analysis. **Chaeheon Woo**: conceptualization, writing – original draft, formal analysis. **Yeongjin Kim**: conceptualization, writing – original draft, formal analysis. **Kyung In Kim**: formal analysis. **Donghyeon Lee**: formal analysis. **Minjae Kim**: data curation. **Jae‐Young Choi**: conceptualization, writing – review and editing, supervision, funding acquisition.

## Conflicts of Interest

The authors declare no conflicts of interest.

## Supporting information




**Supporting File**: smll75004‐sup‐0001‐SuppMat.docx.

## Data Availability

The data that support the findings of this study are available from the corresponding author upon reasonable request.
