# Supplementary material for: Thermo‐Kinetic Design of Magnesiothermic Reduction for Morphology‐Retaining Porous Si@C Anodes
Source: Small. 2026 Jul 31;22(55):e75004. doi: 10.1002/smll.75004 (PMC13630405; doi:10.1002/smll.75004)
Supplement: Supplementary file 1 [file SMLL-22-e75004-s001.docx]

Supporting Information

Thermo-Kinetic Design of Magnesiothermic Reduction for Morphology-Retaining Porous Si@C Anodes

*Chaeheon Woo^a‡^, Yeongjin Kim^a‡^, Xue Dong^b^, Byung Joo Jeong^a^, Xiaojie Zhang^a^, Hyeon-Seok Bang^a,d,e^, Kyung In Kim^a^, Minjae Kim^a^, Donghyeon Lee^a^, Junyoung Mun^a,e,f*^, Hak Ki Yu^c*^, and Jae-Young Choi^a,d,e*^*

**Figure S1.** (a) Photographs of Mg powder (Mg-P) and Mg turnings (Mg-T). (b) N_2_ adsorption-desorption isotherms of the Mg sources.

**Figure S2.** (a) SEM image of an agglomerated region observed for Mg-P_700_. (b) SEM–EDS elemental maps (Si, O, and Mg) acquired from the same area.

**Table S1.** Mass changes obtained from TGA profiles of carbon-coated samples prepared at different coating temperatures, quantified over the temperature windows assigned to Si oxidation (200–350 °C) and carbon combustion (480–650 °C).

|  | **Mass change (%)** | |
| --- | --- | --- |
|  | 200–350 °C (Si oxidation) | 480–650 °C (Carbon combustion) |
| 450 °C | 5.8 | 4.76 |
| 550 °C | 4.1 | -1.92 |
| 650 °C | 1.58 | -7.95 |
| 750 °C | 1.12 | -8.97 |
| 850 °C | 1.82 | -14.83 |

**Table S2.** Inverse resistance values obtained from powder electrical measurements of caron-coated samples as a function of coating temperature.

| **Coating temperature (°C)** | **Inverse resistance (Ω^−1^)** |
| --- | --- |
| 450 | Not measurable |
| 550 | 8.82 x 10**^−6^** |
| 650 | 1.47 x 10**^−2^** |
| 750 | 5.53 x 10**^−2^** |
| 850 | 3.38 x 10**^−1^** |

**Figure S3.** SEM images of the carbon-coated products prepared at different coating temperatures: (a–e) before etching and (f–j) after etching for samples coated at 450, 550, 650, 750, and 850 °C, respectively.

**Table S3.** Textural properties of the samples before and after acid etching.

|  | **Before acid etching** | **After acid etching** |
| --- | --- | --- |
| BET value (m^2^ g^−1^) | 28.24 | 235.22 |
| BJH pore volume (cm^3^ g^−1^) | 0.0997 | 0.8331 |
| Average pore size (nm) | 16.63 | 14.33 |

**Figure S4.** Tap-density measurement of the Mg-T_700_ powder. A powder mass of 2 g occupied a tapped volume of 4.9 mL, corresponding to an apparent tap density of 0.41 g cm^−3^.

**Figure S5.** TGA/DSC profiles of the final Si@C product measured in air for estimating the apparent Si content.

**Figure S6.** (a) SEM image of the Mg-T_700_-RP sample after vacuum drying, showing a compressed morphology. (b) N_2_ adsorption-desorption isotherms comparing Mg-T_700_ and Mg-T_700_-RP.

**Figure S7.** Cycle-dependent CV curves of (a) Mg-P_700_ and (b) Mg-T_700_-RP.

**Table S4**. Comparison of representative porous Si-based anodes prepared by magnesiothermic reduction, aluminothermic reduction, and selective alloy-dealloying routes.

(Note: N.R. indicates that the corresponding value was not clearly reported in the reference.)

| **Synthesis category** | **Final product** | **Reversible capacity**  **(@n cycles)**  **[mAh g^−1^]** | **Capacity retention**  **[%]** | **Specific current**  **[mA g^−1^]** | **ICE**  **[%]** | **Ref.** |
| --- | --- | --- | --- | --- | --- | --- |
| Aluminothermic reduction | Porous Si@C | 1880  (@250 cycles) | 89 | 1200 | 79 | [1] |
| Aluminothermic reduction | C-coated macroporous  Si/Al_2_O_3_ composite | ~1500  (@100 cycles) | 86 | ~716 | 85.5 | [2] |
| Dealloying | Porous Si@C | 458.5  (@500 cycles) | 84.8 | 1000 | 82.1 | [3] |
| Dealloying | Porous Si@C | 887.9  (@200 cycles) | 80 | 1000 | N.R. | [4] |
| Dealloying | Porous Si | 1525  (@100 cycles) | N.R. | 1000 | N.R. | [5] |
| Dealloying | Nanoporous Si | ~1005  (@>300 cycles) | N.R. | 1193 | N.R. | [6] |
| Magnesiothermic reduction | Mesoporous Si/C  microspheres | 990  (@1000 cycles) | 90 | 1000 | N.R. | [7] |
| Magnesiothermic reduction | Porous Si nanoparticles  @hollow C nanosphere | ~600  (@600 cycles) | N.R. | 1000 | N.R. | [8] |
| Magnesiothermic reduction | Porous Si@C | 822.72  (@500 cycles) | N.R. | 1000 | N.R. | [9] |
| Magnesiothermic reduction | C-coated mesoporous  Si nanosheets | 2236  (@150 cycles) | 71.5 | 1000 | N.R. | [10] |
| Magnesiothermic reduction | Porous Si/C composites | 1702  (@250 cycles) | N.R. | 1000 | N.R. | [11] |
| Magnesiothermic reduction | C-wrapped porous Si | 1639  (@200 cycles) | 84.5 | 1000 | 66.8 | [12] |
| Magnesiothermic reduction | Porous Si@C | 1571  (@100 cycles) | 65.7 | 1074 | 74.2 | This work |

**Figure S8.** EIS Nyquist plots of Mg-T_700_, Mg-P_700_, and Mg-T_700_-RP after cycling.

**Figure S9.** Cycle-dependent dQ/dV curves of (a) Mg-T_700_, (b) Mg-P_700_, and (c) Mg-T_700_-RP.

**Figure S10.** Scan-rate-dependent CV curves of (a) Mg-T_700_, (b) Mg-P_700_, and (c) Mg-T_700_-RP electrodes at different scan rates. (d) Corresponding log(i)–log(v) plots used to estimate the b-values.

**Figure S11.** Cross-sectional SEM images of Mg-T_700_, Mg-P_700_, and Mg-T_700_-RP electrodes (a-c) before and (d-f) after 100 cycles, showing the relative electrode thickness after cycling.

**Figure S12.** Post-mortem TEM characterization of the cycled electrode after 100 cycles. (a) TEM image of the cycled Si@C electrode. (b) High-resolution TEM image of the particle surface after cycling, with the inset showing the lattice-resolved Si domain. (c) SAED pattern of the cycled sample, consistent with crystalline Si.

**References**

1.	Mishra, K.; Zheng, J.; Patel, R.; Estevez, L.; Jia, H.; Luo, L.; El-Khoury, P. Z.; Li, X.; Zhou, X.-D.; Zhang, J.-G., *Electrochimica Acta* **2018,** *269*, 509–516.

2.	Choi, S.; Bok, T.; Ryu, J.; Lee, J.-I.; Cho, J.; Park, S., *Nano Energy* **2015,** *12*, 161–168.

3.	Xin, Y.; Li, Y.; Zhang, Y.; Zhang, F.; He, B.; Tian, H., *Energy & Fuels* **2025,** *39* (15), 7564–7575.

4.	Hoseini, S. M. H.; Adeli, M.; Mahanipour, H. G.; Mahdikhah, V., *Journal of Physics and Chemistry of Solids* **2025**, 113160.

5.	Tao, Y.; Zeng, G.; Xiao, C.; Liu, Y.; Qian, Y.; Feng, J., *Journal of colloid and interface science* **2019,** *554*, 674–681.

6.	Pathak, A. D.; Chanda, U. K.; Samanta, K.; Mandal, A.; Sahu, K. K.; Pati, S., *Electrochimica Acta* **2019,** *317*, 654–662.

7.	Xu, Z.-L.; Gang, Y.; Garakani, M. A.; Abouali, S.; Huang, J.-Q.; Kim, J.-K., *Journal of Materials Chemistry A* **2016,** *4* (16), 6098–6106.

8.	Guo, S.; Hu, X.; Hou, Y.; Wen, Z., *ACS applied materials & interfaces* **2017,** *9* (48), 42084–42092.

9.	Liu, Q.; Ji, Y.; Yin, X.; Li, J.; Liu, Y.; Hu, X.; Wen, Z., *Energy Storage Materials* **2022,** *46*, 384–393.

10.	Qin, G.; Jia, Z.; Sun, S.; Wu, H.; Hu, K.; Liu, D.; Gao, Y.; Chen, J., *ACS Applied Nano Materials* **2024,** *7* (7), 7595–7604.

11.	Su, H.; Li, X.; Liu, C.; Shang, Y.; Liu, H., *Chemical Engineering Journal* **2023,** *451*, 138394.

12.	Gao, P.; Tang, H.; Xing, A.; Bao, Z., *Electrochimica Acta* **2017,** *228*, 545–552
